# Thoracic Paravertebral Block, Multimodal Analgesia, and Monitored Anesthesia Care for Breast Cancer Surgery in Primary Lateral Sclerosis

**DOI:** 10.1155/2016/6301358

**Published:** 2016-04-21

**Authors:** Anis Dizdarevic, Anthony Fernandes

**Affiliations:** Anesthesiology and Pain Management, Columbia University Medical Center, 622 West 168th Street, PH 5, New York, NY 10032, USA

## Abstract

*Objective.* Primary lateral sclerosis (PLS) is a rare idiopathic neurodegenerative disorder affecting upper motor neurons and characterized by spasticity, muscle weakness, and bulbar involvement. It can sometimes mimic early stage of more common and fatal amyotrophic lateral sclerosis (ALS). Surgical patients with a history of neurodegenerative disorders, including PLS, may be at increased risk for general anesthesia related ventilatory depression and postoperative respiratory complications, abnormal response to muscle relaxants, and sensitivity to opioids, sedatives, and local anesthetics. We present a case of a patient with PLS and recent diagnosis of breast cancer who underwent a simple mastectomy surgery uneventfully under an ultrasound guided thoracic paravertebral block, multimodal analgesia, and monitored anesthesia care. Patient reported minimal to no pain or discomfort in the postoperative period and received no opioids for pain management before being discharged home. In patients with PLS, thoracic paravertebral block and multimodal analgesia can provide reliable anesthesia and effective analgesia for breast surgery with avoidance of potential risks associated with general anesthesia, muscle paralysis, and opioid use.

## 1. Introduction

Primary lateral sclerosis (PLS) is a rare degenerative disorder of upper motor neuron function, characterized by progressive spasticity and weakness and affecting the legs, trunk, arms, and bulbar muscles [1]. One of the major clinical challenges in PLS diagnosis is distinguishing it from the more common and fatal ALS, hereditary spastic paraparesis, and other neurodegenerative conditions that may present in similar way early in their course [2]. Progression of neuromuscular degeneration may lead to muscle weakness, atrophy, bulbar dysfunction, muscle denervation, and respiratory compromise (as seen more commonly in ALS), resulting ultimately in aspiration risk, respiratory failure, and death [3]. Patients with neuromuscular disorders presenting for surgery pose an increased risk for surgery and anesthesia related complications and should be very carefully assessed perioperatively [4]. Impairment of respiration, muscle weakness, altered response to muscle relaxants, and aspiration risk may affect safe anesthetic management and postoperative care.

This is the first reported case in English literature of a patient with PLS who underwent a successful mastectomy surgery for breast cancer under an ultrasound guided thoracic paravertebral block, multimodal analgesia, and monitored anesthesia care.

Patient reviewed the case report and gave written permission for the authors to publish the report.

## 2. Case Report

A 64-year-old woman with a history of PLS was scheduled for a left breast simple mastectomy with axillary level I lymph node dissection for recently diagnosed ductal carcinoma in situ. The patient's PLS symptoms included significant deconditioning and generalized muscle weakness requiring wheelchair use, generalized muscle spasms, and dysarthria. At the time of surgery, the patient had no home oxygen requirement. Her documented peak cough flow rate (measure of respiratory muscle weakness) was 70 liters per minute (indicating ineffective cough). Her comorbidities included an increased body mass index (29.9) and hypertension. Preoperatively, the patient's heart rate was 106 beats per minute, blood pressure 143/73, respiratory rate 20 breaths per minute, and oxygen saturation 95% on room air. EKG and chest XR were within normal limits. No pulmonary function tests were available prior to surgery. The patient's pulmonologist and primary medical doctor strongly recommended avoidance of general anesthesia for the surgery due to concerns about increased risks of prolonged mechanical ventilation and postoperative respiratory complications related to her condition. During the preoperative visit, the patient refused general anesthesia and requested a minimally invasive approach. After discussing with the patient and surgery team all the risks and potential complications associated with general anesthesia, including respiratory depression, postoperative mechanical ventilation, and pulmonary complications, the patient's medical condition, anesthetic requirements for the procedure and alternatives, plan was made to proceed with a peripheral nerve block and multimodal monitored anesthesia care. Discussion was also held regarding general anesthesia not being used as a backup plan and aborting the procedure in case of inadequate anesthesia or patient discomfort.

**Figure 1:**
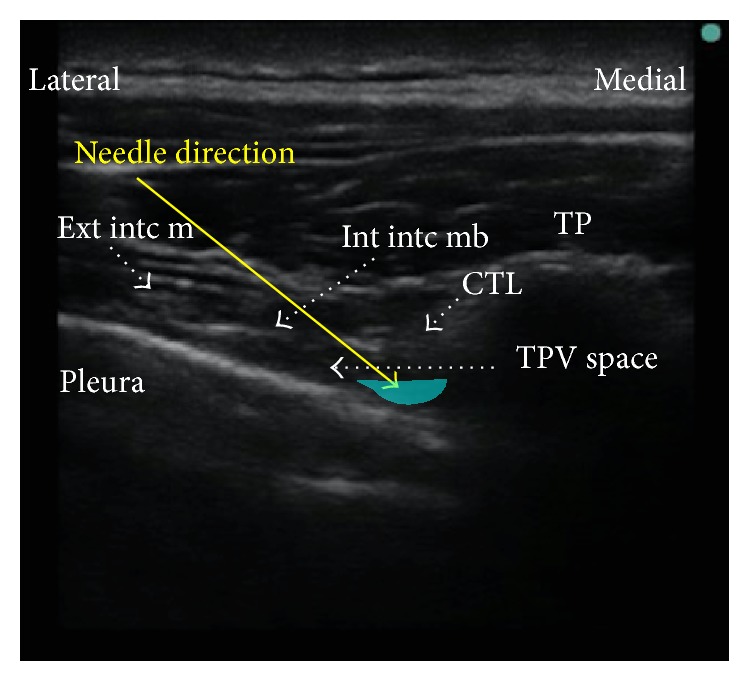
Thoracic paravertebral block, transverse in line technique. Needle trajectory: lateral to medial. TP = transverse process. TPV = thoracic paravertebral. Int into mb = internal intercostal membrane. Ext into m = external intercostal muscle. CTL = costotransverse ligament.

For the thoracic paravertebral block placement, the patient was positioned sitting upright with the arms resting on a Mayo table for support. Standard American Society of Anesthesiologists monitors and supplemental oxygen via nasal cannula were placed. Intravenous midazolam, 1 mg, and fentanyl, 25 mcg, were administered, prior to starting the procedure. A SonoSite M-Turbo high frequency linear transducer (6–15 MHz) probe was placed transverse to the thoracic vertebral column, parallel to the ribs. Under direct ultrasound guidance, a 21-gauge 100 mm Pajunk Sono TAP needle was inserted parallel to the rib, in a lateral to medial direction, in the plane of the ultrasound beam, and directed toward the paravertebral space between the internal intercostal membrane and pleura (Figure 1). Three single shot left-sided paravertebral blocks were performed at the levels T3, T4, and T5 using a total of 35 mL of 0.5% preservative-free ropivacaine with 6 mg of dexamethasone, to extend the duration of the block. A dexmedetomidine infusion (0.4 mcg/kg/hr) was started, along with additional midazolam, 0.5 mg, fentanyl 25 mcg, and ketamine 10 mg for monitored anesthesia care sedation. Prior to the surgical start, cold temperature sensation was assessed by alcohol swab, demonstrating T3 to T5 dermatome coverage. The patient reported mild discomfort with the surgical incision and received additional 10 mL of 1% lidocaine and 0.25% bupivacaine local anesthetic infiltration into the incision area to reinforce the block. A large elliptical incision was made, and the dissection was performed superiorly to 2 cm below the clavicle, medially to the lateral border of the sternum, inferiorly to the level of the inframammary crease, and laterally to the latissimus muscle. The breast tissue was removed off the pectoralis major and serratus anterior muscles. The patient tolerated the entire surgery reporting no discomfort or pain and remained hemodynamically stable, with no significant change in respiratory rate or oxygen saturation. The pain score on PACU arrival was 0/10 on a numeric rating scale. Vital signs were as follows: heart rate 104, blood pressure 110/78, respiratory rate 19, and oxygen saturation of 98% on 3l nasal cannula. Postoperatively the patient received acetaminophen for pain control with a maximal pain score reported as 2/10 and was discharged home on postoperative day 2. The patient did not require any postoperative opioids.

## 3. Discussion

PLS is a rare, idiopathic, slowly progressive, nonfamilial, neurodegenerative disorder of the upper motor neuron affecting corticospinal and corticobulbar tracts in the arms, legs, and face. The most common clinical presentation of PLS includes spasticity, hyperreflexia, and mild muscle weakness. Bulbar symptoms can include dysarthria, dysphagia, and emotional lability. The pathophysiologic basis remains unknown, and there is currently no definitive diagnosis or disease marker. Imaging and laboratory testing are used to rule out other diagnoses including metabolic, physiologic, and anatomic confounding processes [5]. PLS classically is described as a pure upper motor neuron neurodegenerative disorder. Newer electrophysiological data may suggest that some patients with PLS may also have concurrent lower motor neuron involvement, similar to amyotrophic lateral sclerosis, ALS, more common and fatal condition [6]. Marked weakness can be absent but patients can progress to debilitating spasticity and have respiratory compromise. It is argued that PLS may be a slowly progressive form of ALS, with subsequent lower motor neuron involvement. A small number of ALS patients initially present with pure upper motor neuron findings but most develop lower motor neuron signs and EMG findings within 4 years [7]. The progression may even occur over several decades. Currently, there is very sparse literature pertaining to the anesthetic management and risks involving patients with PLS. Given some similarities in clinical presentation and pathophysiology between PLS and other neurodegenerative disorders, our discussion of anesthetic management could herein be extrapolated to include those patient populations as well.

The extent of muscle weakness, respiratory dysfunction, and bulbar involvement have been of special importance in patients presenting for surgical procedures. Respiratory complications are a major cause of morbidity and mortality in patients with neurodegenerative conditions. Pure PLS does not involve the same severity and progression of respiratory dysfunction as seen in ALS, but, nevertheless, is of concern [3, 8]. Respiratory failure can result from inability to clear secretions, carbon dioxide retention, and compromised ventilator function. Bulbar muscle dysfunction with tongue fasciculation and pharyngeal muscle weakness may lead to an increased risk of aspiration and related complications [9]. Preoperative pulmonary assessment with pulmonary function tests, if deemed necessary, would be useful in determining a degree of pulmonary dysfunction and potential risk of postoperative respiratory failure. Furthermore, in neurodegenerative conditions, after denervation and prolonged immobilization, upregulation of acetylcholine receptors occurs at the neuromuscular junction and along the skeletal muscle membranes. Administration of depolarizing neuromuscular blockers, such as succinylcholine, can lead to activation of an unpredictably large quantity of receptors resulting in an abnormally high efflux of potassium. Extubation readiness may be difficult to assess as a result of baseline muscle weakness and altered pulmonary function. The intraoperative use of nondepolarizing muscle relaxants should be very carefully weighed against the potential increased likelihood of prolonged weakness and postoperative ventilatory support. Patients with neuromuscular disease, including PLS, may also be sensitive to sedative and analgesic agents, and their judicious use is recommended [10].

Intraoperative recommendations for anesthetic management of patients with PLS and similar neurodegenerative disorders would include the use of rapid reversible short-acting anesthetic and analgesic agents and avoiding (depolarizing) and/or minimizing (nondepolarizing) muscle paralysis. Postoperatively, conservative dosing of opioids and sedatives, enhanced monitoring with continuous pulse oximetry, and use of multimodal analgesia to optimize pain management are recommended.

Regional anesthesia, including local infiltration and peripheral and neuraxial nerve blocks, has been successfully used in patients with neuromuscular disorders to avoid possible respiratory and other complications associated with general anesthesia and opioid use [11, 12]. Agnoletti et al.'s description of a thoracic paravertebral block (TPVB) for breast surgery in a patient with ALS is the only report similar to ours published in the English literature [13]. The authors performed a classic approach to paravertebral blocks at the thoracic T1–T3 levels, using a 3 mL solution of 0.75% ropivacaine at each level.

Paravertebral block can offer specific advantages for patients with neuromuscular disorders, especially when general anesthesia may be unfavorable or relatively contraindicated. By administering local anesthetic near the target somatic nerve roots, unilateral anesthesia and analgesia can be achieved without bilateral sympathectomy and associated side effects. Furthermore, PVB allows for efficient spontaneous breathing and early mobilization, minimizing the risk of postoperative respiratory dysfunction. Schnabel et al. and Tahiri et al., in their meta-analysis, evaluated the efficacy and safety of TPVB in breast surgery and also compared thoracic PVB to general anesthesia [14, 15]. Their results demonstrated that TPVB, combined with sedation, provided effective surgical anesthesia for patients undergoing breast procedures. They also concluded that TPVB alone, or in addition to general anesthesia, provided better postoperative pain control with little adverse effects, when compared to other analgesic options. In addition, when used instead of general anesthesia, TPVB resulted in pain scores that were significantly decreased postoperatively. The potential adverse effects associated with TPVB were Horner's syndrome (most common) and rare cases of accidental intravascular injection of part of the local anesthetic, pneumothorax, epidural spread, hypotension, and intercostal nerve trauma.

In our case, the patient received additional local anesthetic infiltration at the incision site prior to the start of surgery. This was expected for two potential reasons: (1) cutaneous innervation of the anterior chest wall via intercostal nerves may take longer to become anesthetized when using regional technique and (2) the middle supraclavicular nerve, originating from the C3 and C4 cervical nerves, also supplies the skin over the pectoralis major muscle and is not anesthetized with the paravertebral block. We added dexamethasone to the local anesthetic to prolong the analgesic duration of the block [16]. We also added subanesthetic dose of ketamine to our multimodal regiment as it has been reported effective in reducing opioid requirements postoperatively with mild to no adverse effects [17]. Sympathomimetic effects on the cardiovascular system, especially in the presence of dexmedetomidine, would be expected to be minimal, as we observed in our case. Finally, as another part of our multimodal analgesia regiment but also as the main anesthetic in monitored anesthesia care, we chose dexmedetomidine. Dexmedetomidine has been reported to reduce the intra- and postoperative opioid consumption, postoperative pain, and nausea and vomiting, with no effect on the time of recovery [18]. Furthermore, dexmedetomidine, when used for sedation in monitored anesthesia care, has been shown to provide greater hemodynamic stability and less respiratory depression when compared to propofol as well as placebo with rescue midazolam and fentanyl, with the most common side effect of intraoperative bradycardia [19, 20].

## 4. Conclusion

Patients with primary lateral sclerosis and other similar neurodegenerative disorders presenting for surgery may be at increased risk for surgery and anesthesia related complications and should be carefully assessed perioperatively by all healthcare professionals involved in their care. Special emphasis should be paid to respiratory function, aspiration risk, and muscle dysfunction. Thoracic paravertebral block, along with multimodal analgesia, can provide reliable anesthesia and effective analgesia for breast surgery, with avoidance of potential risks associated with general anesthesia, muscle paralysis, and opioid use.
